# Transplantation Treatment of Extensive Soft-Tissue Defects in Lower Extremities with a Combination of Cross-Bridge Flap and Combined Free-Tissue Flap Covered by Vacuum Sealing Drainage: One Case Report

**DOI:** 10.2174/1874325001711010704

**Published:** 2017-07-31

**Authors:** Xiaohua Pan, Guangyao Wang, Tun Hing Lui

**Affiliations:** 1 Department of orthopedic and traumatology, Shenzhen BaoAn People Hospital affiliated Southern Medical University, Shenzhen, Guangdong 518101, People’s Republic of China; 2 Department of orthopedic and traumatology, Panyu District hospital of traditional Chinese medicine, Guangzhou, Guangdong 510515,People’s Republic of China; 3 Department of Orthopaedic and Traumatology, North District hospital, Sheung Shui, Hong Kong

**Keywords:** Vacuum sealing drainage, Soft tissue defect, Cross leg flap, Free tissue flap, Transplantation, Combined fracture, Tibia

## Abstract

This article reported the ultilization of cross-bridge flap transplantation and combined free-tissue flap transplantation to treat a 54-year-old male with Gustilo type III-C injuries. Thorough debridement, external fixation and vacuum sealing drainage were performed in the fist-stage treatment. After the removal of negative pressure on VSD devices, the joined free-tissue flaps and the cross-bridge flap were performed to repair the extensive soft-tissue defects. One month later the pedicle of cross-bridge flap was divided and the external fixator connecting both the lower legs was removed. In 3-month follow-up, the extensive defects was completely covered by a nearly normal skin and radiograph showed tibia and talus healing.

## 1 INTRODUCTION

With the increase in traffic, severe injuries of lower extremities caused by traffic accident and daily accidents happen more frequently than before [1]. Due to the thin texture of soft tissues and low ductility of skin in lower extremities, these injuries usually lead to the extensive soft-tissue defects with severe open comminuted fractures, infection, and injuries of nerves and main vessels, which consist mostly of Gustilo type III-C injuries [2]. The management of the extensive soft-tissue defects with open comminuted fracture has long been a great therapeutic challenge for orthopedic surgeons.

Free-tissue flap transplantation has been used to treat the soft-tissue defects of lower extremities. These flaps are often harvested from different locations of the body, such as thoracic umbilical flap, scapular flap, anterolateral thigh flap and so on [3].However, on the one hand, isolated free-tissue flap transplantation cannot effectively cover extensive soft-tissue defects due to the limited size of the transferred flap. On the other hand, it cannot manage to cure soft-tissue defects without suitable vessel for anastomosis with the transferred flap. Cross-bridge pedicled flap, which can provide a temporary blood supply for the transferred tissue, has been usually used to treat the soft-tissue defects without the suitable vessel for anastomosis with ipsilateral free flap [4]. However, either free-tissue flap or cross-bridge pedicled flap cannot repair the extensive soft-tissue defects individually [5, 6].

In the present report, we described a patient at the risk of amputation, who was successfully treated with an innovative method. This method not only makes full use of the advantages but also avoids the disadvantages of both kinds of flap transplantation; namely, free-tissue flap transplantation and cross-bridge flap transplantation.

## 2 CASE REPORT

A 54-year-old male had his right lower leg injured by a motorcycle wheel in a traffic accident, which led to the disfunction of the wounded leg with continuously intense pain. Bone, tendon, vessels and nerves were exposed with extensive soft-tissue defects of the wounded leg (Fig. **1a**). Radiographs showed tibial comminuted fracture, talar open fracture and subluxation of right ankle (Figs. **1b**-**c**).

**Fig. (2):**
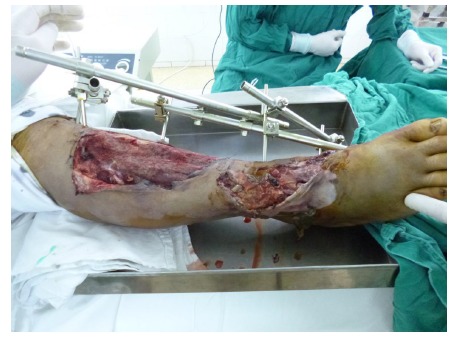
An external fixator was applied to stabilize the fractures. There was an extensive soft tissue defect with sluggish circulation.

**Fig. (3):**
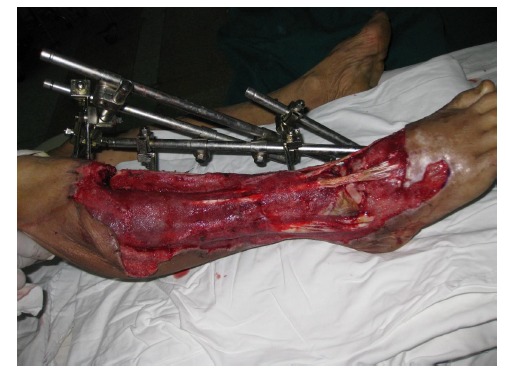
Healthy granulation tissue surrounding the soft-tissue defects after repeated debridement and VSD.

One hour after admission, emergency operation was performed in the condition of general anesthesia. The necrotic tissue was completely debrided in the first place. After primary debridement, the talar fracture was fixed with two 5mm screws. The dislocation of ankle joint was properly corrected. The comminuted proximal tibial fracture was immobilized by an external fixator and there was a dull-red coloured wound of 40cm ×10cm, with deep tissue exposed, necrotic tissue and faint yellow purulent secretions (Fig. **2**). Direct anastomosis of anterior tibial artery, its accompanying vein and perforating branch of the peroneal artery were performed separately. The wounds were covered with two VSD sponges, sealed by two semipermeable membranes of 20cm ×10cm in size. The negative pressure was provided by negative drainage bag. About 2000 ml of blood was lost pre-operatively and intra-operatively. Moreover, the VSD led to the further decrease in the level of hemochrome in the blood of patient. Supportive and symptomatic treatment was performed after emergency operation. After three days of operation, second-time debridement and VSD were performed. Another three debridement operations and VSD were performed at the seventh, fourteenth, and thirtieth day separately. At this moment, fresh granulation tissue surrounded the soft-tissue defects (Fig. **3**).

Free-tissue flap transplantation surgery was personally designed based on the position, size and severity of soft tissue defects and the general condition of patient. A free anterolateral thigh perforator flap and a free thoracic umbilical flap were designed (Fig. **4a**). The blood stream of cutaneous branches of inferior epigastric artery and lateral femoral circumflex artery nourishing the designed flaps was detected by Doppler ultrasound; whilst, the free thoracic umbilical flap pedicled with inferior epigastric vessels and ascending branch of lateral femoral circumflex vessels were performed to reconstruct the bone and soft-tissue defects in the wounded lower leg. Both the pedicles of vessels were of 10 cm. The designed thoracic umbilical flap pedicled with inferior epigastric vessels was harvested (Fig. **4b**). The anastomosis between the transversal branch of lateral femoral circumflex artery and inferior epigastric artery and the anastomosis of accompanying veins of both arteries described above were performed by microsurgical technique. The anterolateral thigh perforator flap was sutured with thoracic umbilical flap to make a combined free-tissue flap (Fig. **4c**), which was boarding into the donor area of anterolateral thigh perforator flap on the left leg temporarily (Fig. **4d**). Two days later, the combined free-tissue flap was designed to recover the affected area of skin defects (Fig. **5a**). The blood stream of posterior tibial artery nourishing the designed cross-bridge pedicled flap of the contralateral leg was detected by Doppler ultrasound; whilst, the cross-bridge pedicled flap was harvested to provide blood supply to the combined free-tissue flap from the posterior tibial artery (Fig. **5b**). The cross-fixation of the bilateral lower legs by external fixator was performed to make them immobilized as an integral. After this performance, the vessels were anastomosed. The artery of combined free-tissue flap was sutured with the posterior tibial artery of cross-bridge flap. The anastomosis between the stem vein of combined free-tissue flap and the great saphenous vein of cross-bridge flap and the anostomosis between the branch veins of combined free-tissue flap and accompanying veins of posterior tibial artery of cross-bridge flap were performed at the same time (Fig. **5c**). The combined free-tissue flap covered the soft-tissue defects and was sutured with the soft tissue of the recipient site. Following this performance, the donor area of the upper leg was veiled by VSD.

**Fig. (6):**
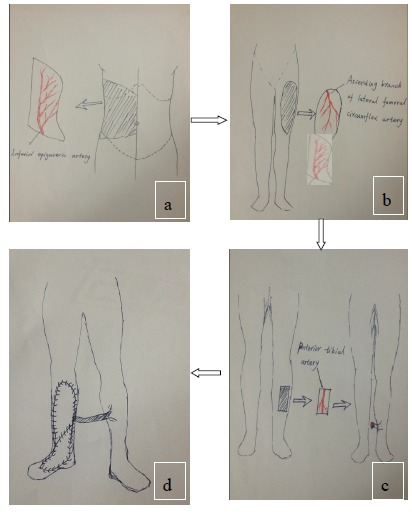
Diagrammatic sketch of the combined free-tissue flap The design and harvest of combined flap consisted of anterolateral thigh perforator flap and thoracic umbilical flap (**a**: the harvest of thoracic umbilical flap, **b**: the harvested anterolateral thigh perforator flap, **c**: the harvest of cross-leg flap, **d**:the combined free flap covering the injured site with anastomosis with the cross-leg flap).

The pedicle of cross-bridge flap was divided one month after the cross-bridge flap transplantation. Moreover, the external fixator, which immobilized both the lower legs, was removed and the external fixator of wounded lower leg was still retained. At 3-month follow-up, both the lower legs had a nearly normal appearance with complete coverage and radiographs of the wounded lower leg showed tibia and talus healing (Figs. **6a**-**b**, **c**).

## 3 DISCUSSION

For orthopedic surgeon, it is a big challenge to decide how many flaps should and can be excised and how to cover and restore soft-tissue defects when they perform the large area limb salvages. The strategy of two-stage repair is regarded as a more reliable treatment for soft-tissue defects of the lower leg [7].However, for patient with extensive soft-tissue defects coupled with severe comminuted fractures, it is a more reliable treatment to apply the strategy of multiple-stage repair, which is very necessary for those severe wounds without suitable vessel for anastomosis. We applied damage control orthopedics for this patient and handled severe associated injuries in the first place. Therefore, we focused on performing wide debridement of necrotic tissues, maintaining vital signs stable, preventing wound infections, and making simple external fixation for fractures in the first-stage treatment. Finally vacuum sealing drainage (VSD) was used in the treatment of wound infection of the soft-tissue in the wounded lower leg. After VSD treatment, the infection of wound was effectively controlled and the condition of the wound greatly improved. This negative pressure technique played an important role in the protection of large wound in the lower leg by controlling infection, stimulating growth of granulation, improving wound’s condition, reducing exposure of deep tissues and decreasing the size of flap transferred for soft-tissue defects [8]. Although thorough and timely debridement was performed and preventive antibiotic drugs were applied, the exposed bone and soft tissues were still under the risk of infection. Therefore, the temporary coverage by VSD is an indispensable part of the treatment protocols [9].

In this case, we reported that the transplantation of cross-bridge flap was performed to provide temporary blood supply for the transferred combined free-tissue flaps due to lack of suitable vessels for anastomosis with ipsilateral free flap in the wounded leg. This solution is the only method to be applied when none of the recipient vessels for anastomosis is available and if patient wants to avoid the operation of amputation. One of disadvantages of cross-bridge flap transplantation is the long-term external fixation of both the lower legs, which requires the patient to stay in bed during this period. This is very harmful to those patients with poor general condition. Therefore, the indication for cross-bridge flap transplantation should be strictly limited because one of the main arteries in the donor site of blood vessels would be sacrificed, which is very risky when the operation fails. However, it is impossible to repair extensive soft-tissue defects with a cross-bridge flap individually. Therefore, we applied the constructive method of combining the cross-bridge flap transplantation and combined free-tissue flap transplantation to repair the extensive soft-tissue defects with severe injuries in the main arteries.

## CONCLUSION

Combined cross-leg and free flaps can be useful in management of large soft tissue defect associated with severe lower leg injuries.

## Figures and Tables

**Fig.(1a):**
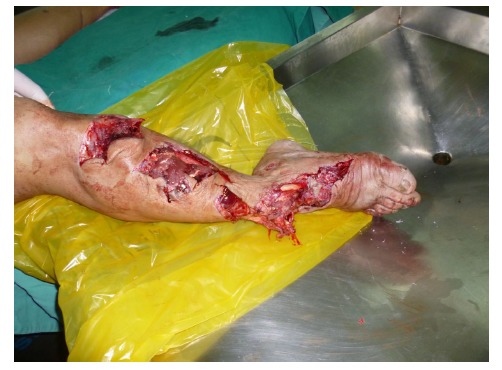
Extensive soft-tissue defects of the right lower leg and foot before debridement, accompanied by comminuted fracture of the proximal tibia, spiral fracture of the proximal fibula, fracture of the talus, and dislocation of the ankle joint.(**a**:clinical photo, **b**:x-ray of the leg, **c**: x-ray of the ankle).

**Fig. (1b):**
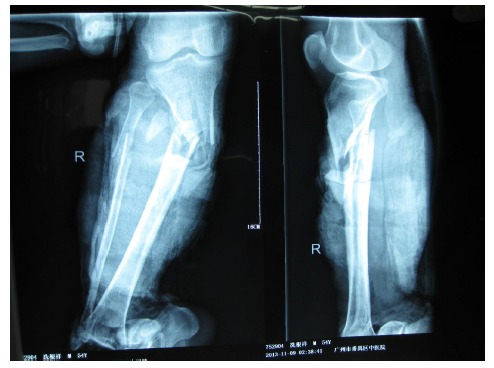
Extensive soft-tissue defects of the right lower leg and foot before debridement, accompanied by comminuted fracture of the proximal tibia, spiral fracture of the proximal fibula, fracture of the talus, and dislocation of the ankle joint.(**a**:clinical photo, **b**:x-ray of the leg, **c**: x-ray of the ankle).

**Fig. (1c):**
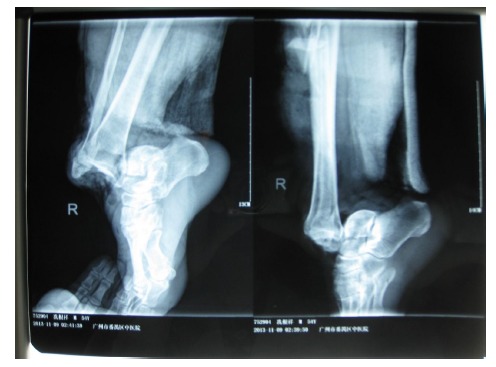
Extensive soft-tissue defects of the right lower leg and foot before debridement, accompanied by comminuted fracture of the proximal tibia, spiral fracture of the proximal fibula, fracture of the talus, and dislocation of the ankle joint.(**a**:clinical photo, **b**:x-ray of the leg, **c**: x-ray of the ankle).

**Fig. (4a):**
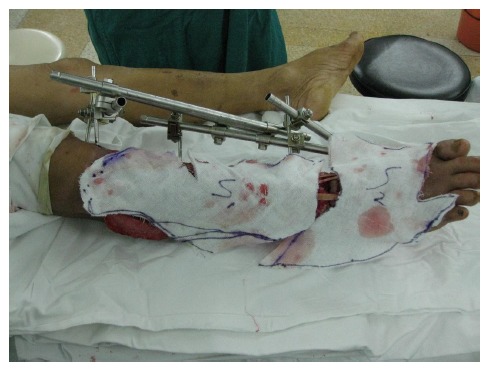
The design and harvest of combined flap consisted of anterolateral thigh perforator flap and thoracic umbilical flap (**a**: the design of the combined flap, **b**:the harvest of thoracic umbilical flap, **c**: the harvested combined flap, **d**:the combined flap was temporarily sutured to the donated area of anterolateral thigh perforator flap).

**Fig. (4b):**
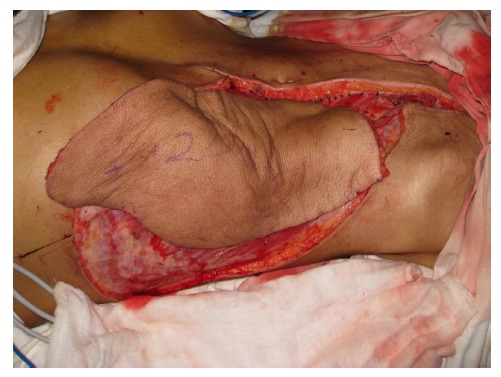
The design and harvest of combined flap consisted of anterolateral thigh perforator flap and thoracic umbilical flap (**a**: the design of the combined flap, **b**:the harvest of thoracic umbilical flap, **c**: the harvested combined flap, **d**:the combined flap was temporarily sutured to the donated area of anterolateral thigh perforator flap).

**Fig. (4c):**
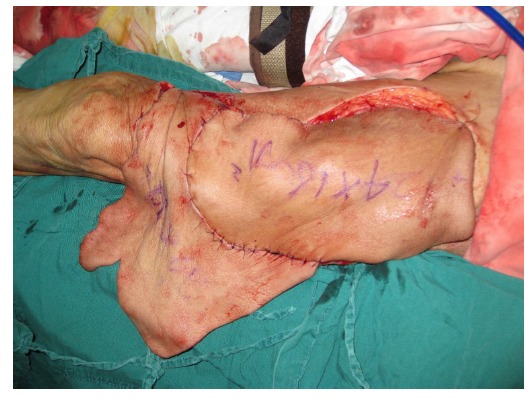
The design and harvest of combined flap consisted of anterolateral thigh perforator flap and thoracic umbilical flap (**a**: the design of the combined flap, **b**:the harvest of thoracic umbilical flap, **c**: the harvested combined flap, **d**:the combined flap was temporarily sutured to the donated area of anterolateral thigh perforator flap).

**Fig. (4d):**
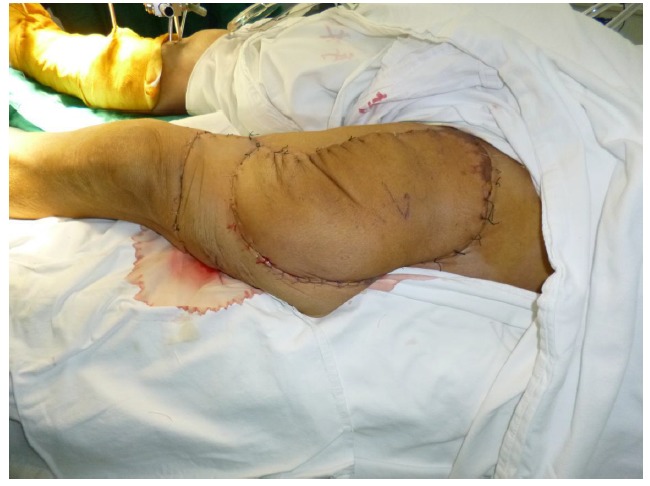
The design and harvest of combined flap consisted of anterolateral thigh perforator flap and thoracic umbilical flap (**a**: the design of the combined flap, **b**:the harvest of thoracic umbilical flap, **c**: the harvested combined flap, **d**:the combined flap was temporarily sutured to the donated area of anterolateral thigh perforator flap).

**Fig. (5a):**
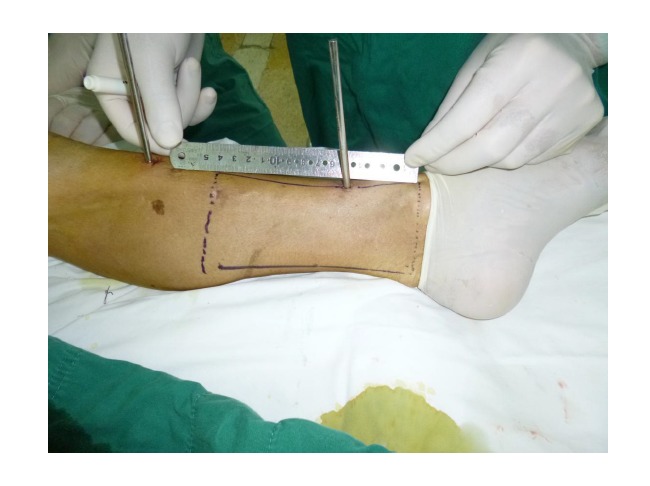
The procedure of cross-leg flap transplantation (**a**: the design of cross-leg flap, **b**: the harvest of cross-leg flap, **c**: the cross-leg external fixation and the anostomosis between cross-bride flap and combined flap).

**Fig. (5b):**
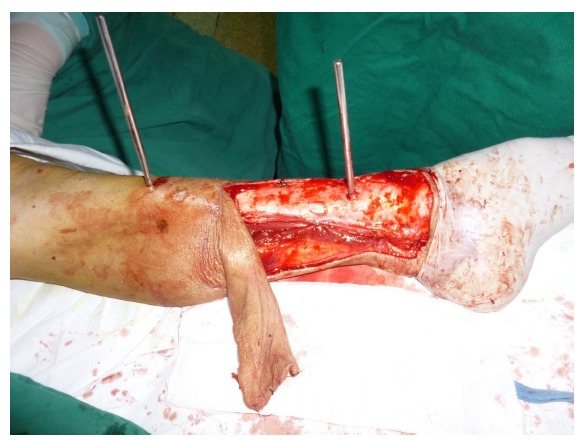
The procedure of cross-leg flap transplantation (**a**: the design of cross-leg flap, **b**: the harvest of cross-leg flap, **c**: the cross-leg external fixation and the anostomosis between cross-bride flap and combined flap).

**Fig. (5c):**
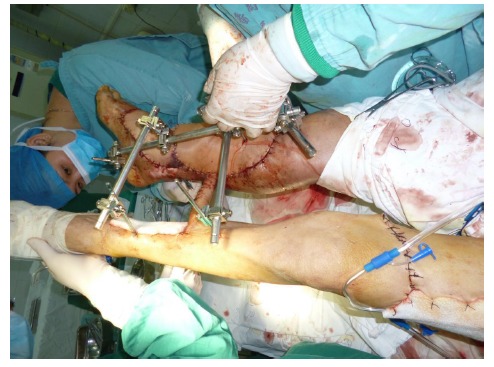
The procedure of cross-leg flap transplantation (**a**: the design of cross-leg flap, **b**: the harvest of cross-leg flap, **c**: the cross-leg external fixation and the anostomosis between cross-bride flap and combined flap).

## LIST OF ABBREVIATION

VSD: = Vacuum Sealing Drainage
